# 1-Bromo-2-[(*E*)-2-nitro­ethen­yl]benzene

**DOI:** 10.1107/S1600536811050963

**Published:** 2011-11-30

**Authors:** Pei-Hua Zhao, Zhan-Heng Feng, Mei Zhang, Ya-Qing Liu, Gui-Zhe Zhao

**Affiliations:** a Research Center for Engineering Technology of Polymeric Composites of Shanxi Province, College of Materials Science and Engineering, North University of China, Taiyuan 030051, People’s Republic of China; b College of Chemsitry, Nankai University, Tianjin 300071, People’s Republic of China

## Abstract

In the title compound, C_8_H_6_BrNO_2_, the dihedral angle between the planes of the benzene ring and the nitro group is 22.99 (12)°. In the crystal, inversion dimers associated by pairs of short Br⋯O contacts [3.2319 (17) Å] occur.

## Related literature

For background to nitro-olefins and their synthetic applications, see: Barret & Graboski (1986 ▶); Berner *et al.* (2002 ▶); Ballini *et al.* (1992 ▶).
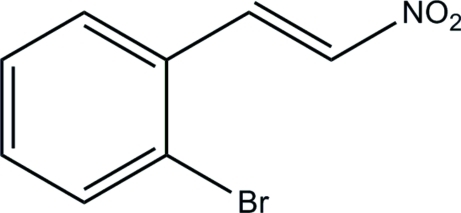


## Experimental

### 

#### Crystal data

C_8_H_6_BrNO_2_
*M* *_r_* = 228.05
Monoclinic, 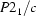

*a* = 6.9570 (18) Å
*b* = 15.646 (2) Å
*c* = 7.9470 (13) Å
β = 109.336 (5)°
*V* = 816.2 (3) Å^3^
*Z* = 4
Mo *K*α radiation
μ = 4.99 mm^−1^
*T* = 113 K
0.20 × 0.18 × 0.16 mm

#### Data collection

Rigaku Saturn724 CCD diffractometer
Absorption correction: multi-scan (*CrystalClear*; Rigaku/MSC, 2005 ▶) *T*_min_ = 0.435, *T*_max_ = 0.502
10346 measured reflections
1945 independent reflections
1466 reflections with *I* > 2σ(*I*)
*R*_int_ = 0.042

#### Refinement

*R*[*F* ^2^ > 2σ(*F* ^2^)] = 0.024
*wR*(*F* ^2^) = 0.056
*S* = 1.08
1945 reflections
109 parameters
H-atom parameters constrained
Δρ_max_ = 0.33 e Å^−3^
Δρ_min_ = −0.78 e Å^−3^

### 

Data collection: *CrystalClear* (Rigaku/MSC, 2005 ▶); cell refinement: *CrystalClear*; data reduction: *CrystalClear*; program(s) used to solve structure: *SHELXS97* (Sheldrick, 2008 ▶); program(s) used to refine structure: *SHELXL97* (Sheldrick, 2008 ▶); molecular graphics: *SHELXTL* (Sheldrick, 2008 ▶); software used to prepare material for publication: *CrystalStructure* (Rigaku/MSC, 2005 ▶).

## Supplementary Material

Crystal structure: contains datablock(s) global, I. DOI: 10.1107/S1600536811050963/hb6539sup1.cif

Structure factors: contains datablock(s) I. DOI: 10.1107/S1600536811050963/hb6539Isup2.hkl

Supplementary material file. DOI: 10.1107/S1600536811050963/hb6539Isup3.cml

Additional supplementary materials:  crystallographic information; 3D view; checkCIF report

## Figures and Tables

**Fig. 1.**
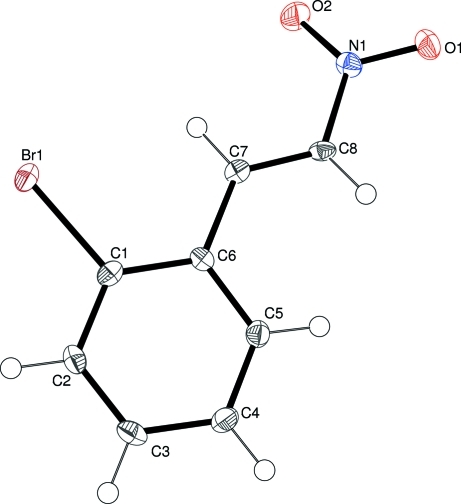
The molecular structure of (I). Displacement ellipsoids are drawn at the 30% probability level and H atoms are shown as small spheres of arbitrary radii.

**Fig. 2.**
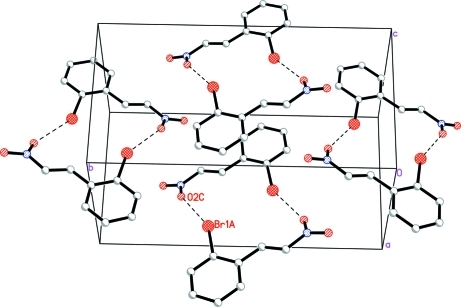
The crystal packing for (I).

## supplementary crystallographic information

### Comment

Nitro-olefins are useful building blocks in organic synthesis (Barret *et al.*, 1986). Furthermore, the charater of these compounds as electron-deficient alkenes allows easy 1,4-addtion reactions and this opens the way to synthetically useful C—C and C—*X* (*X* = N, O) bond-forming reactions (Berner *et al.* (2002), Ballini *et al.* (1992)). The title compound, (I), belongs to the class of fuctionalized nitroolefins.

As shown in Fig. 1, the dihedral angle between carbon double bond and phenyl groups is 12.2 (2) °. As shown in Fig. 2, the crystal packing shows the weak O···Br intermolecular interactions.

### Experimental

2-Bromobenzaladehyde (39.8 mmol, 7.36 g), nitromethane (99.2 mmol, 5.38 ml), and methanol (16.80 ml) are added to a 3-neck round bottomed flask and cooled to zero degree centigrade. While maintaining the internal reaction temperature between zero and ten degrees centigrade, aqueous 1*M* NaOH (100.2 mmol, 100.20 ml) is added by an additon funnel and the mixture is stirred for 15 min. Ice water mixture (70.00 ml) is added and the reaction is stirred at zero degree centigrade for 30 min. The reaction mixture is slowly added to aqueous 8*M* HCl (536.0 mmol, 67.00 ml) and allowed to stir until the rection is confirmed complete by TLC. The reaction mixture is filtered and recrystallized from ethanol to give the product.  Colourless prisms of (I) were obtained by slow evaporation of the dichloromethane/n-hexane solutions at room temperature.

### Refinement

All the H atoms were positioned geometrically (C—H = 0.95 Å) and refined as riding with *U*_iso_(H) = 1.2*U*_eq_(C).

### Crystal data

**Table:**

C_8_H_6_BrNO_2_	*F*(000) = 448
*M**_r_* = 228.05	*D*_x_ = 1.856 Mg m^−^^3^
Monoclinic, *P*2_1_/*c*	Mo *K*α radiation, λ = 0.71073 Å
*a* = 6.9570 (18) Å	Cell parameters from 2970 reflections
*b* = 15.646 (2) Å	θ = 2.6–28.0°
*c* = 7.9470 (13) Å	µ = 4.99 mm^−^^1^
β = 109.336 (5)°	*T* = 113 K
*V* = 816.2 (3) Å^3^	Prism, colorless
*Z* = 4	0.20 × 0.18 × 0.16 mm

### Data collection

**Table:**

Rigaku Saturn724 CCD diffractometer	1945 independent reflections
Radiation source: rotating anode	1466 reflections with *I* > 2σ(*I*)
multilayer	*R*_int_ = 0.042
Detector resolution: 14.22 pixels mm^-1^	θ_max_ = 27.8°, θ_min_ = 2.6°
ω and φ scans	*h* = −9→9
Absorption correction: multi-scan (*CrystalClear*; Rigaku/MSC, 2005)	*k* = −20→20
*T*_min_ = 0.435, *T*_max_ = 0.502	*l* = −10→10
10346 measured reflections	

### Refinement

**Table:**

Refinement on *F*^2^	Primary atom site location: structure-invariant direct methods
Least-squares matrix: full	Secondary atom site location: difference Fourier map
*R*[*F*^2^ > 2σ(*F*^2^)] = 0.024	Hydrogen site location: inferred from neighbouring sites
*wR*(*F*^2^) = 0.056	H-atom parameters constrained
*S* = 1.08	*w* = 1/[σ^2^(*F*_o_^2^) + (0.020*P*)^2^] where *P* = (*F*_o_^2^ + 2*F*_c_^2^)/3
1945 reflections	(Δ/σ)_max_ = 0.003
109 parameters	Δρ_max_ = 0.33 e Å^−^^3^
0 restraints	Δρ_min_ = −0.78 e Å^−^^3^

### Special details

**Table:**

Geometry. All e.s.d.'s (except the e.s.d. in the dihedral angle between two l.s. planes) are estimated using the full covariance matrix. The cell e.s.d.'s are taken into account individually in the estimation of e.s.d.'s in distances, angles and torsion angles; correlations between e.s.d.'s in cell parameters are only used when they are defined by crystal symmetry. An approximate (isotropic) treatment of cell e.s.d.'s is used for estimating e.s.d.'s involving l.s. planes.
Refinement. Refinement of *F*^2^ against ALL reflections. The weighted *R*-factor *wR* and goodness of fit *S* are based on *F*^2^, conventional *R*-factors *R* are based on *F*, with *F* set to zero for negative *F*^2^. The threshold expression of *F*^2^ > σ(*F*^2^) is used only for calculating *R*-factors(gt) *etc*. and is not relevant to the choice of reflections for refinement. *R*-factors based on *F*^2^ are statistically about twice as large as those based on *F*, and *R*- factors based on ALL data will be even larger.

### Fractional atomic coordinates and isotropic or equivalent isotropic displacement parameters (Å^2^)

**Table:**

	*x*	*y*	*z*	*U*_iso_*/*U*_eq_	
Br1	0.39712 (4)	0.607142 (14)	0.81215 (3)	0.02051 (8)	
O1	0.1113 (2)	0.21839 (10)	0.6270 (2)	0.0218 (4)	
O2	0.3362 (2)	0.29119 (10)	0.8336 (2)	0.0238 (4)	
N1	0.2150 (3)	0.28344 (11)	0.6807 (2)	0.0153 (4)	
C1	0.3043 (3)	0.58570 (14)	0.5620 (3)	0.0141 (5)	
C2	0.2914 (3)	0.65593 (14)	0.4508 (3)	0.0153 (5)	
H2	0.3316	0.7109	0.5007	0.018*	
C3	0.2203 (3)	0.64552 (14)	0.2684 (3)	0.0179 (5)	
H3	0.2093	0.6935	0.1925	0.021*	
C4	0.1644 (3)	0.56451 (15)	0.1954 (3)	0.0190 (5)	
H4	0.1156	0.5571	0.0696	0.023*	
C5	0.1804 (3)	0.49484 (14)	0.3071 (3)	0.0160 (5)	
H5	0.1438	0.4398	0.2561	0.019*	
C6	0.2490 (3)	0.50341 (13)	0.4930 (3)	0.0119 (4)	
C7	0.2672 (3)	0.42903 (13)	0.6100 (3)	0.0132 (5)	
H7	0.3405	0.4360	0.7334	0.016*	
C8	0.1882 (3)	0.35266 (13)	0.5549 (3)	0.0135 (5)	
H8	0.1138	0.3435	0.4324	0.016*	

### Atomic displacement parameters (Å^2^)

**Table:**

	*U*^11^	*U*^22^	*U*^33^	*U*^12^	*U*^13^	*U*^23^
Br1	0.02929 (14)	0.01733 (14)	0.01372 (13)	−0.00098 (11)	0.00552 (10)	−0.00406 (9)
O1	0.0270 (10)	0.0133 (8)	0.0233 (9)	−0.0033 (7)	0.0058 (8)	0.0004 (7)
O2	0.0272 (10)	0.0228 (9)	0.0148 (9)	0.0010 (7)	−0.0020 (7)	0.0036 (7)
N1	0.0178 (10)	0.0113 (10)	0.0182 (10)	0.0032 (8)	0.0079 (8)	0.0012 (8)
C1	0.0107 (11)	0.0190 (12)	0.0120 (11)	0.0007 (9)	0.0032 (9)	−0.0014 (9)
C2	0.0151 (12)	0.0119 (12)	0.0207 (13)	−0.0022 (9)	0.0081 (10)	−0.0021 (9)
C3	0.0178 (12)	0.0158 (12)	0.0207 (13)	−0.0014 (10)	0.0074 (10)	0.0057 (10)
C4	0.0211 (13)	0.0227 (14)	0.0122 (12)	−0.0046 (10)	0.0042 (10)	−0.0008 (10)
C5	0.0173 (12)	0.0146 (12)	0.0161 (12)	−0.0010 (9)	0.0056 (10)	−0.0023 (9)
C6	0.0099 (11)	0.0113 (11)	0.0148 (12)	0.0014 (8)	0.0046 (9)	0.0015 (9)
C7	0.0119 (11)	0.0150 (12)	0.0124 (11)	0.0032 (9)	0.0037 (9)	0.0005 (9)
C8	0.0164 (12)	0.0132 (12)	0.0111 (12)	0.0047 (9)	0.0050 (9)	0.0037 (9)

### Geometric parameters (Å, °)

**Table:**

Br1—C1	1.906 (2)	C3—H3	0.9500
O1—N1	1.239 (2)	C4—C5	1.387 (3)
O2—N1	1.234 (2)	C4—H4	0.9500
N1—C8	1.444 (3)	C5—C6	1.401 (3)
C1—C2	1.394 (3)	C5—H5	0.9500
C1—C6	1.402 (3)	C6—C7	1.468 (3)
C2—C3	1.377 (3)	C7—C8	1.328 (3)
C2—H2	0.9500	C7—H7	0.9500
C3—C4	1.395 (3)	C8—H8	0.9500
			
O2—N1—O1	123.39 (19)	C3—C4—H4	120.1
O2—N1—C8	119.86 (18)	C4—C5—C6	121.8 (2)
O1—N1—C8	116.75 (18)	C4—C5—H5	119.1
C2—C1—C6	121.6 (2)	C6—C5—H5	119.1
C2—C1—Br1	116.81 (16)	C1—C6—C5	116.97 (19)
C6—C1—Br1	121.57 (16)	C1—C6—C7	121.70 (19)
C3—C2—C1	119.9 (2)	C5—C6—C7	121.31 (19)
C3—C2—H2	120.0	C8—C7—C6	124.4 (2)
C1—C2—H2	120.0	C8—C7—H7	117.8
C2—C3—C4	119.9 (2)	C6—C7—H7	117.8
C2—C3—H3	120.0	C7—C8—N1	120.14 (19)
C4—C3—H3	120.0	C7—C8—H8	119.9
C5—C4—C3	119.7 (2)	N1—C8—H8	119.9
C5—C4—H4	120.1		
			
C6—C1—C2—C3	0.9 (3)	Br1—C1—C6—C7	−2.4 (3)
Br1—C1—C2—C3	−178.25 (16)	C4—C5—C6—C1	−1.0 (3)
C1—C2—C3—C4	−1.0 (3)	C4—C5—C6—C7	−179.4 (2)
C2—C3—C4—C5	0.2 (3)	C1—C6—C7—C8	168.8 (2)
C3—C4—C5—C6	0.9 (4)	C5—C6—C7—C8	−13.0 (3)
C2—C1—C6—C5	0.1 (3)	C6—C7—C8—N1	179.9 (2)
Br1—C1—C6—C5	179.22 (16)	O2—N1—C8—C7	−10.9 (3)
C2—C1—C6—C7	178.5 (2)	O1—N1—C8—C7	168.8 (2)
